# Supplementary material for: Association of urinary excretion rates of uric acid with biomarkers of kidney injury in patients with advanced chronic kidney disease
Source: PLoS One. 2024 Jun 11;19(6):e0304105. doi: 10.1371/journal.pone.0304105 (PMC11166352; doi:10.1371/journal.pone.0304105)
Supplement: S2 File — (DOCX) [file pone.0304105.s002.docx]

NOTA: *Se realiza el análisis con los n=109 pacientes que tienen datos en todos los marcadores de daño tubular y renal crónico, salvo DKK (se dispone de datos en 104 de los 109 pacientes)*

**Tabla 1. Descripción de los niveles séricos de ácido úrico y sus marcadores de excreción renal.**

|  | n | Media | DT | Mediana | P25 | P75 | Mín | Máx |
| --- | --- | --- | --- | --- | --- | --- | --- | --- |
| Úrico basal (mg/dL) | 109 | 7.6 | 1.7 | 7.6 | 6.5 | 8.3 | 3.4 | 13.0 |
| Úrico en orina de 24 h basal (mg/24 h) | 109 | 277.8 | 138.6 | 260.0 | 160.0 | 350.0 | 80.0 | 680.0 |
| Concentración de úrico en orina basal (mg/dL) | 109 | 13.79 | 6.41 | 12.78 | 8.99 | 17.37 | 4.09 | 31.67 |
| Aclaramiento de ácido úrico basal (mL/m) | 109 | 2.6 | 1.2 | 2.3 | 1.7 | 3.3 | .8 | 5.7 |
| Excreción fraccionada de úrico basal | 108 | 9.73 | 4.09 | 8.61 | 6.82 | 11.40 | 2.80 | 22.25 |

**Tabla 2. Descripción de los marcadores de daño tubular y renal crónico.**

|  | n | Media | DT | Mediana | P25 | P75 | Mín | Máx |
| --- | --- | --- | --- | --- | --- | --- | --- | --- |
| Urico basal (mg/dL) | 109 | 7.6 | 1.7 | 7.6 | 6.5 | 8.3 | 3.4 | 13.0 |
| DKK3 en orina basal (ng/ml) | 104 | 1.64 | 1.59 | 1.34 | .39 | 2.23 | .01 | 8.01 |
| NGAL en orina basal (ng/mL) | 109 | 32.93 | 42.83 | 15.69 | 5.28 | 47.80 | 2.47 | 303.00 |
| KIM-1 en orina basal (pg/ml) | 109 | 1364.9 | 1182.2 | 1025.9 | 492.6 | 1955.8 | 82.1 | 6348.8 |
| Interleukina 1B en orina (pg/mL) | 109 | 152.17 | 93.89 | 145.93 | 81.64 | 205.21 | 5.21 | 450.93 |
| MCP basal en orina (pg/mL) | 109 | 205.22 | 166.44 | 151.19 | 88.09 | 281.03 | 10.07 | 739.95 |

**Tabla 3. Correlación (Rho de Spearman) entre los niveles séricos de ácido úrico o marcadores de excreción y los marcadores de daño tubular y renal crónico.**

|  | | **DKK3 en orina basal (ng/ml)** | **NGAL en orina basal (ng/mL)** | **KIM-1 en orina basal (pg/ml)** | **Interleukina 1B en orina (pg/mL)** | **MCP basal en orina (pg/mL)** |
| --- | --- | --- | --- | --- | --- | --- |
| **Urico basal (mg/dL)** | Rho | -.057 | .076 | .044 | -.129 | .075 |
|  | p | .564 | .430 | .646 | .180 | .441 |
|  | N | 104 | 109 | 109 | 109 | 109 |
| **Uirco en orina de 24 h basal (mg/24 h)** | Rho | .139 | .163 | .063 | -.035 | .040 |
|  | p | .161 | .091 | .517 | .719 | .679 |
|  | N | 104 | 109 | 109 | 109 | 109 |
| **Concentración de urico en orina basal (mg/dL)** | Rho | .159 | .156 | .143 | -.038 | .087 |
|  | p | .108 | .106 | .137 | .697 | .370 |
|  | N | 104 | 109 | 109 | 109 | 109 |
| **Aclaramiento de ácido úrico basal (mL/m)** | Rho | .188 | .165 | .067 | -.006 | .048 |
|  | p | .056 | .086 | .486 | .949 | .619 |
|  | N | 104 | 109 | 109 | 109 | 109 |
| **Excreción fraccionada de urico basal** | Rho | **.429** | **.364** | .147 | -.141 | .103 |
|  | p | .000 | .000 | .128 | .146 | .291 |
|  | N | 103 | 108 | 108 | 108 | 108 |

**Tabla 4. Comparación de los niveles de marcadores de daño tubular y renal crónico según niveles séricos de úrico y diferentes marcadores de excreción renal.**

|  | **Úrico basal (mg/dL) (cuartiles)** | | | | | | | | | | | |  |
| --- | --- | --- | --- | --- | --- | --- | --- | --- | --- | --- | --- | --- | --- |
|  | **<= 6.4** | | | **6.5 - 7.6** | | | **7.7 - 8.3** | | | **8.4+** | | |  |
|  | **Media** | **DT** | **Mediana** | **Media** | **DT** | **Mediana** | **Media** | **DT** | **Mediana** | **Media** | **DT** | **Mediana** | **p^*^** |
| DKK3 en orina basal (ng/ml) | 2.15 | 1.80 | 1.84 | 1.22 | 1.09 | .95 | 1.65 | 1.73 | 1.41 | 1.63 | 1.65 | 1.05 | 0.374 |
| NGAL en orina basal (ng/mL) | 25.47 | 23.64 | 13.75 | 33.37 | 59.84 | 8.62 | 39.49 | 46.75 | 21.87 | 33.37 | 30.87 | 28.19 | 0.551 |
| KIM-1 en orina basal (pg/ml) | 1367.4 | 1040.5 | 1347.9 | 1228.4 | 1171.5 | 865.6 | 1523.5 | 1275.7 | 968.5 | 1349.9 | 1277.8 | 1114.2 | 0.699 |
| Interleukina 1B en orina (pg/mL) | 150.93 | 87.57 | 143.79 | 165.74 | 101.98 | 163.79 | 164.84 | 107.26 | 152.36 | 125.16 | 73.09 | 110.93 | 0.473 |
| MCP basal en orina (pg/mL) | 231.77 | 189.00 | 151.19 | 198.02 | 191.16 | 108.61 | 180.50 | 147.91 | 139.84 | 211.36 | 131.65 | 177.02 | 0.394 |

^*^Test de Kruskall-Wallis

|  | **Uirco en orina de 24 h basal (mg/24 h) (cuartiles)** | | | | | | | | | | | |  |
| --- | --- | --- | --- | --- | --- | --- | --- | --- | --- | --- | --- | --- | --- |
|  | **<= 170.0** | | | **171.0 - 260.0** | | | **261.0 - 360.0** | | | **361.0+** | | |  |
|  | **Media** | **DT** | **Mediana** | **Media** | **DT** | **Mediana** | **Media** | **DT** | **Mediana** | **Media** | **DT** | **Mediana** | **p^*^** |
| DKK3 en orina basal (ng/ml) | 1.50 | 1.36 | 1.33 | 1.49 | 1.32 | 1.40 | 1.35 | 1.31 | .84 | 2.28 | 2.19 | 1.69 | 0.377 |
| NGAL en orina basal (ng/mL) | 33.11 | 57.89 | 11.72 | 22.05 | 19.82 | 12.38 | 34.49 | 31.97 | 25.61 | 41.08 | 47.46 | 21.70 | 0.356 |
| KIM-1 en orina basal (pg/ml) | 1248.6 | 1225.8 | 865.6 | 1362.3 | 1330.1 | 1087.2 | 1603.4 | 1083.0 | 1403.2 | 1249.2 | 1116.0 | 917.7 | 0.307 |
| Interleukina 1B en orina (pg/mL) | 160.31 | 101.27 | 144.50 | 159.80 | 76.31 | 167.36 | 117.24 | 76.25 | 99.86 | 173.04 | 110.36 | 154.14 | 0.123 |
| MCP basal en orina (pg/mL) | 188.85 | 176.33 | 139.17 | 230.83 | 196.98 | 168.28 | 218.83 | 142.46 | 167.95 | 186.45 | 152.37 | 146.23 | 0.376 |

^*^Test de Kruskall-Wallis

|  | **Concentración de urico en orina basal (mg/dL) (cuartiles)** | | | | | | | | | | | |  |
| --- | --- | --- | --- | --- | --- | --- | --- | --- | --- | --- | --- | --- | --- |
|  | **<= 9.26** | | | **9.27 - 13.19** | | | **13.20 - 18.42** | | | **18.43+** | | |  |
|  | **Media** | **DT** | **Mediana** | **Media** | **DT** | **Mediana** | **Media** | **DT** | **Mediana** | **Media** | **DT** | **Mediana** | **p^*^** |
| DKK3 en orina basal (ng/ml) | 1.28 | 1.26 | .95 | 1.53 | 1.28 | 1.41 | 1.65 | 1.63 | 1.01 | 2.25 | 2.13 | 1.95 | 0.417 |
| NGAL en orina basal (ng/mL) | 31.42 | 57.00 | 11.12 | 30.04 | 31.80 | 17.13 | 24.54 | 24.58 | 18.84 | 48.88 | 48.85 | 32.51 | 0.264 |
| KIM-1 en orina basal (pg/ml) | 1001.4 | 884.9 | 851.5 | 1615.1 | 1199.0 | 1362.4 | 1219.5 | 1068.9 | 853.2 | 1728.8 | 1500.7 | 1237.6 | 0.070 |
| Interleukina 1B en orina (pg/mL) | 168.10 | 100.78 | 154.86 | 139.68 | 79.38 | 153.07 | 148.50 | 87.15 | 140.21 | 150.68 | 110.52 | 147.36 | 0.816 |
| MCP basal en orina (pg/mL) | 176.80 | 155.27 | 133.85 | 243.83 | 193.78 | 157.88 | 184.90 | 139.98 | 151.19 | 222.59 | 176.37 | 178.03 | 0.364 |

^*^Test de Kruskall-Wallis

|  | **Aclaramiento de ácido úrico basal (mL/m) (cuartiles)** | | | | | | | | | | | |  |
| --- | --- | --- | --- | --- | --- | --- | --- | --- | --- | --- | --- | --- | --- |
|  | **<= 1.7** | | | **1.8 - 2.3** | | | **2.4 - 3.2** | | | **3.3+** | | |  |
|  | **Media** | **DT** | **Mediana** | **Media** | **DT** | **Mediana** | **Media** | **DT** | **Mediana** | **Media** | **DT** | **Mediana** | **p^*^** |
| DKK3 en orina basal (ng/ml) | 1.29 | 1.25 | 1.06 | 1.36 | 1.25 | 1.05 | 1.70 | 1.76 | 1.44 | 2.25 | 1.95 | 1.96 | 0.161 |
| NGAL en orina basal (ng/mL) | 29.34 | 58.74 | 9.71 | 26.44 | 26.42 | 13.21 | 39.63 | 32.58 | 32.73 | 37.92 | 44.86 | 20.00 | 0.112 |
| KIM-1 en orina basal (pg/ml) | 1206.6 | 1108.2 | 865.6 | 1445.2 | 1381.2 | 1156.8 | 1384.8 | 1079.8 | 962.4 | 1427.9 | 1158.9 | 1159.7 | 0.832 |
| Interleukina 1B en orina (pg/mL) | 168.51 | 98.64 | 163.79 | 151.77 | 72.24 | 155.93 | 103.36 | 79.59 | 95.22 | 173.25 | 108.14 | 155.21 | 0.021 |
| MCP basal en orina (pg/mL) | 190.61 | 166.36 | 131.85 | 195.63 | 151.80 | 165.25 | 250.45 | 205.72 | 148.86 | 195.12 | 149.55 | 167.26 | 0.684 |

^*^Test de Kruskall-Wallis

|  | **Excreción fraccionada de urico basal (cuartiles)** | | | | | | | | | | | |  |
| --- | --- | --- | --- | --- | --- | --- | --- | --- | --- | --- | --- | --- | --- |
|  | **<= 6.73** | | | **6.74 - 8.66** | | | **8.67 - 11.94** | | | **11.95+** | | |  |
|  | **Media** | **DT** | **Mediana** | **Media** | **DT** | **Mediana** | **Media** | **DT** | **Mediana** | **Media** | **DT** | **Mediana** | **p^*^** |
| DKK3 en orina basal (ng/ml) | .86 | .80 | **.83** | 1.20 | 1.25 | **.55** | 2.13 | 1.75 | **1.61** | 2.48 | 1.89 | **1.95** | <0.001 |
| NGAL en orina basal (ng/mL) | 26.67 | 61.26 | **5.16** | 23.09 | 27.68 | **11.64** | 35.67 | 38.83 | **26.22** | 49.08 | 37.68 | **46.96** | 0.002 |
| KIM-1 en orina basal (pg/ml) | 1095.3 | 902.9 | 968.5 | 1422.7 | 1235.9 | 853.2 | 1467.2 | 1519.4 | 928.0 | 1497.8 | 976.1 | 1351.3 | 0.458 |
| Interleukina 1B en orina (pg/mL) | 166.76 | 84.63 | 172.36 | 152.10 | 95.47 | 140.21 | 139.97 | 74.33 | 133.43 | 148.05 | 119.18 | 128.07 | 0.535 |
| MCP basal en orina (pg/mL) | 149.55 | 96.36 | 119.23 | 225.96 | 179.72 | 176.00 | 208.97 | 173.52 | 135.18 | 236.72 | 189.97 | 174.33 | 0.343 |

^*^Test de Kruskall-Wallis

**Figure 1. Relationship between fractional excretion of uric acid and markers of renal and tubular damage**.


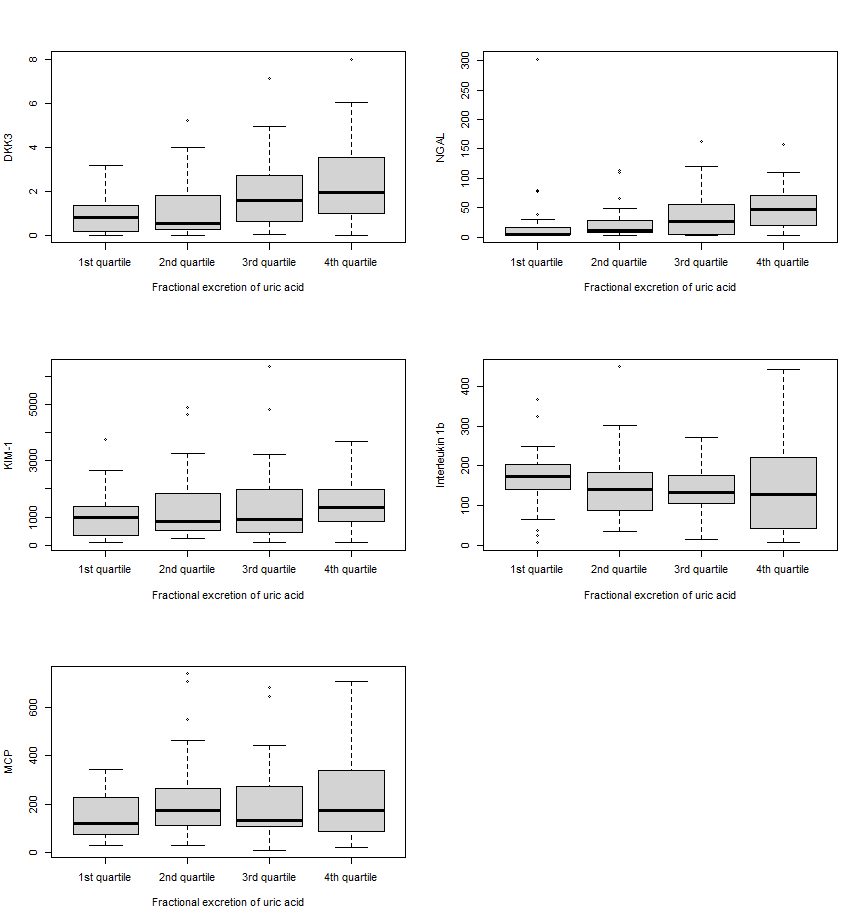


**Figure 2. Smoothing regression showing the relationship between fractional excretion of uric acid and**  **markers of renal and tubular damage**.

| 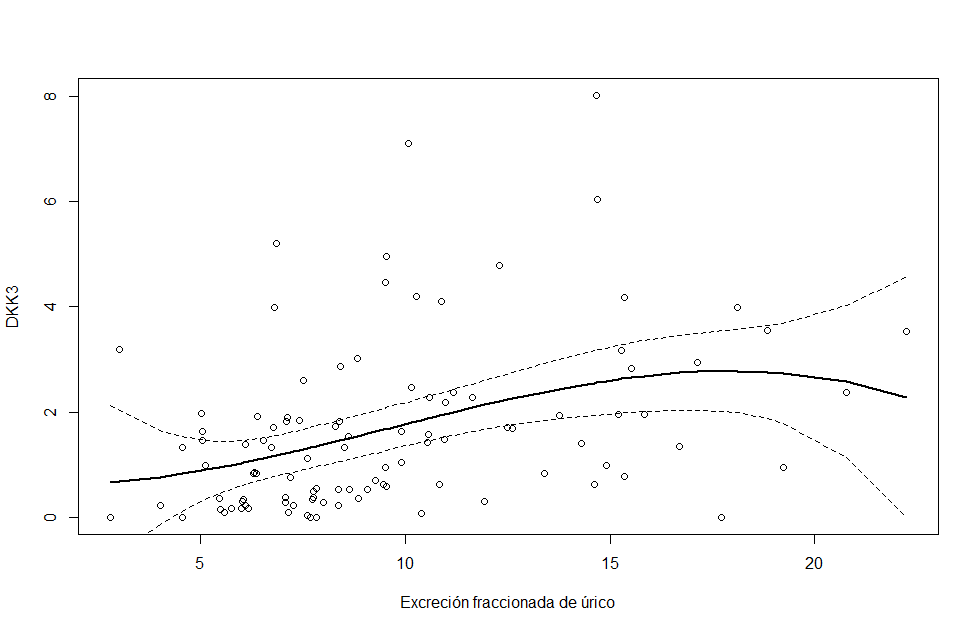 | 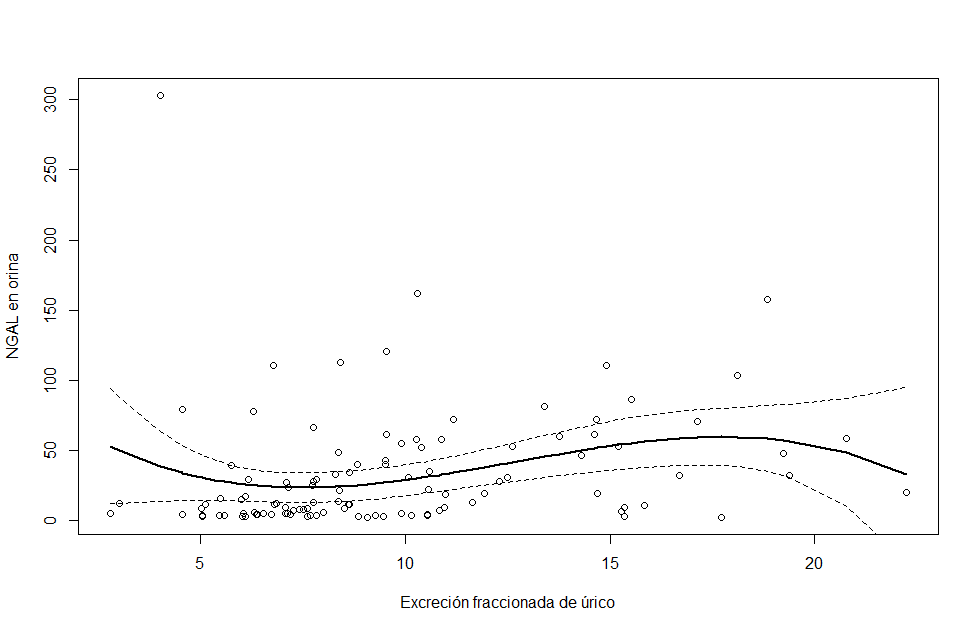 |
| --- | --- |
| 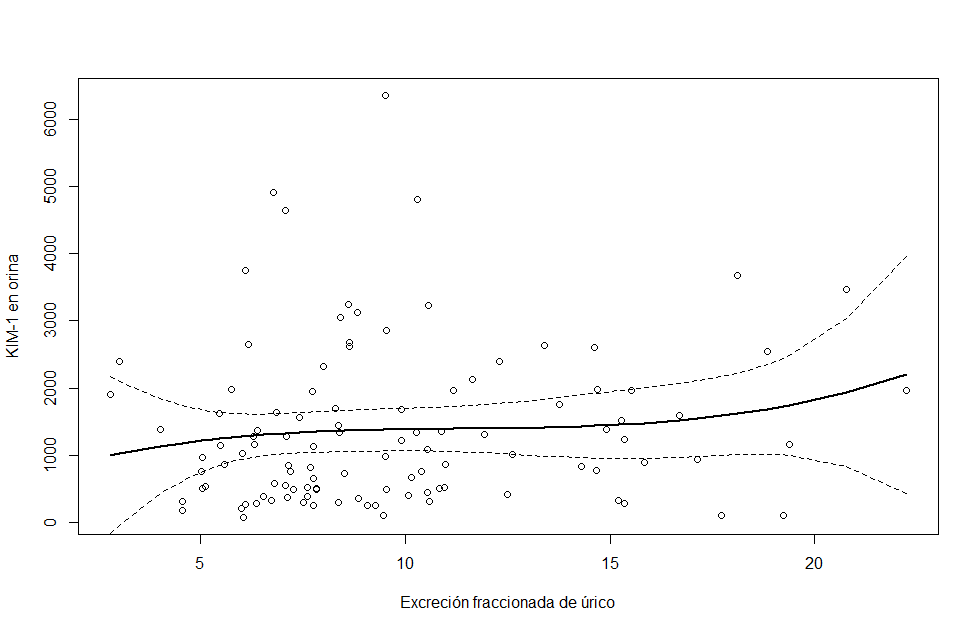 | 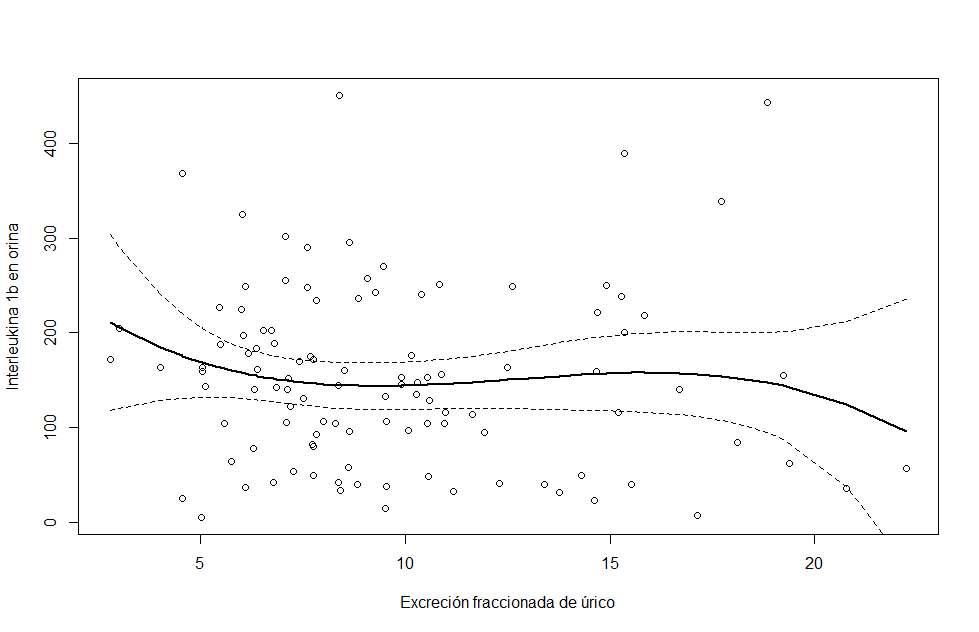 |
| 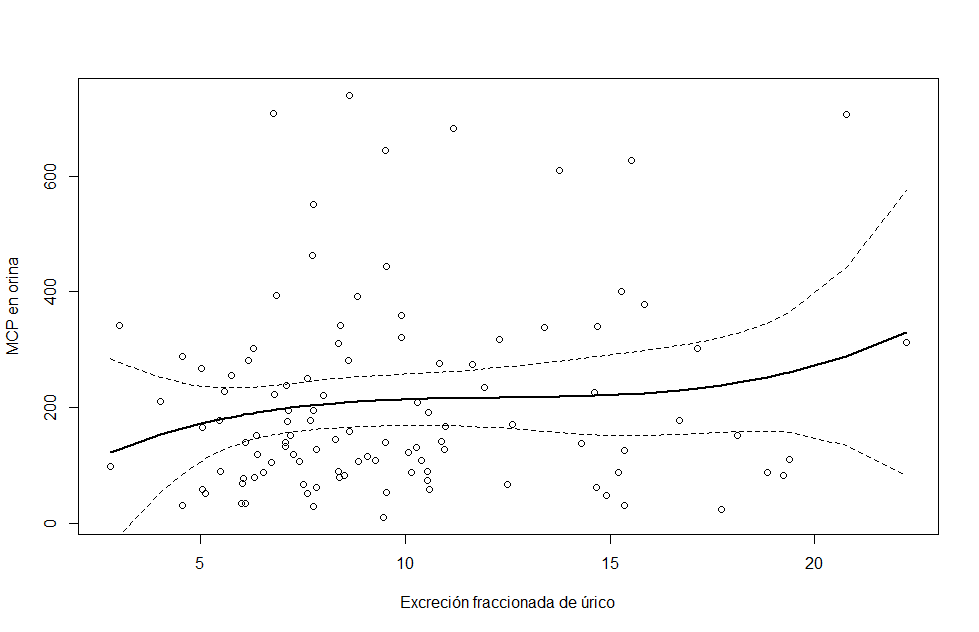 |  |

**Table x. Adjusted effects of fractional excretion of uric acid and levels of markers of renal and tubular damage.**

|  | **DKK3** | | **NGAL** | |
| --- | --- | --- | --- | --- |
|  | **B (95% CI)** | **p** | **B (95% CI)** | **p** |
| **Fractional excretion**  **of uric acid** | 0.14 (0.08;0.21) | <0.001 | 2.04 (0.02;4.06) | 0.048 |
|  |  |  |  |  |
| **Fractional excretion**  **of uric acid** |  |  |  |  |
| 1^st^ quartile | Referencia |  | Referencia |  |
| 2^nd^ quartile | 0.21 (-0.56;0.98) | 0.596 | -5.73 (-29.17;17.7) | 0.628 |
| 3^rd^ quartile | 1.10 (0.30;1.91) | 0.008 | 7.17 (-17.02;31.37) | 0.558 |
| 4^rd^ quartile | 1.61 (0.81;2.41) | <0.001 | 20.31 (-4.12;44.74) | 0.540 |

^*^Ajustado por edad, tratamiento con IECAs y tratamiento con estatinas
