# Supplementary material for: Association of urinary excretion rates of uric acid with biomarkers of kidney injury in patients with advanced chronic kidney disease
Source: PLoS One. 2024 Jun 11;19(6):e0304105. doi: 10.1371/journal.pone.0304105 (PMC11166352; doi:10.1371/journal.pone.0304105)
Supplement: S3 File — (DOCX) [file pone.0304105.s003.docx]

**Statistical analysis**

Data are presented as mean, standard deviation, quartiles or percentages for quantitative and categorical variables, respectively. The distribution of all examined variables was assessed by Komogorov-Smirnov test, histograms, and probability plots.

In the bivariate analysis, the relationship among acid uric levels and markers of renal and tubular damage was assessed by nonparametric Spearman’s rho correlation coefficient. Additionally, markers’ values were compared among quartiles of different uric acid values, using the Kruskall-Wallis test to perform comparisons.

Nonlinear generalized additive regression models (GAM) were then used to study the association of markers of renal and tubular damage with serum uric acid levels or uric acid excretion markers. Variables adjusted for potential confounding included age, … Natural log-transformation was applied to proteinuria due to its skewed distribution. For the non-linear relationships identified, smooth terms were introduced in the models using penalized regression splines. The validity of model assumptions was evaluated using analysis of residuals, and log-transformation of outcomes were applied when necessary.

Scientific Package for Social Science (PASW Statistics for Windows, Version 25.0, Chicago, IL, USA: SPSS Inc.) and R 3.6.3 (R Foundation for Statistical Computing, Vienna, Austria, accessed <https://www.r-project.org/>) software was used for statistical analyses. GAM models were adjusted using the ‘mgdcv’ package. Values of p<0.05 were considered statistically significant.

**Figura. Histograma de la distribución de los marcadores de daño renal estudiados.**


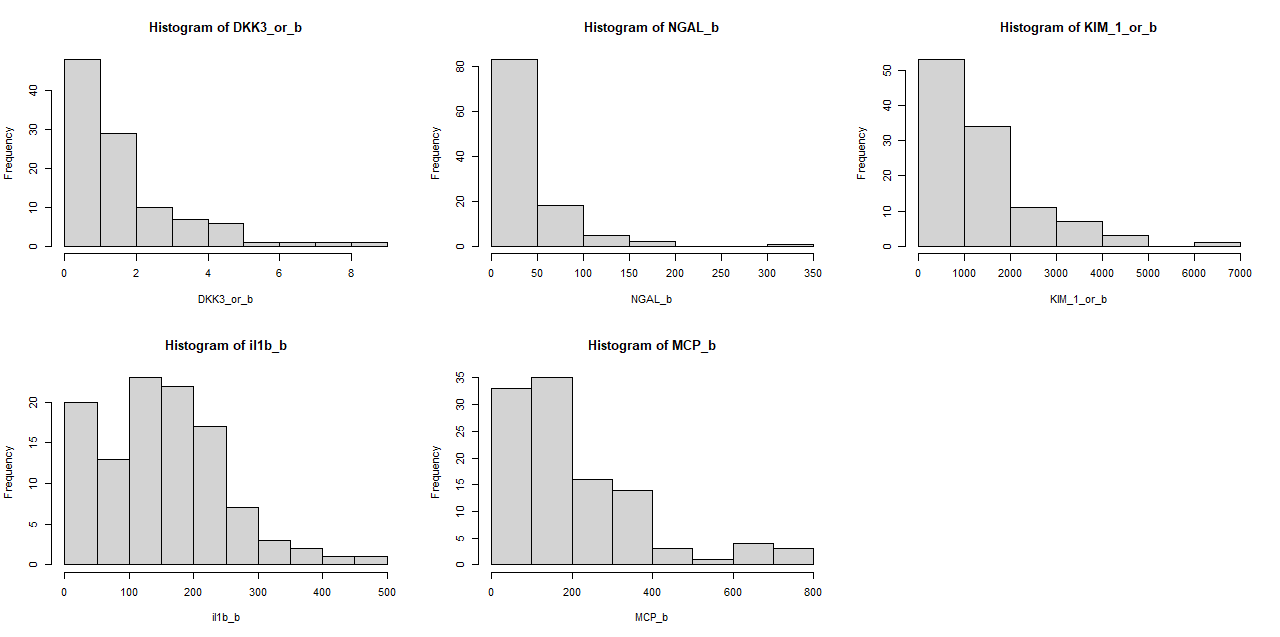


# **ANÁLISIS DEL ÚRICO BASAL**

**FIGURA. Smoothing regression showing the relationship between BASAL URIC ACID and**  **markers of renal and tubular damage**.


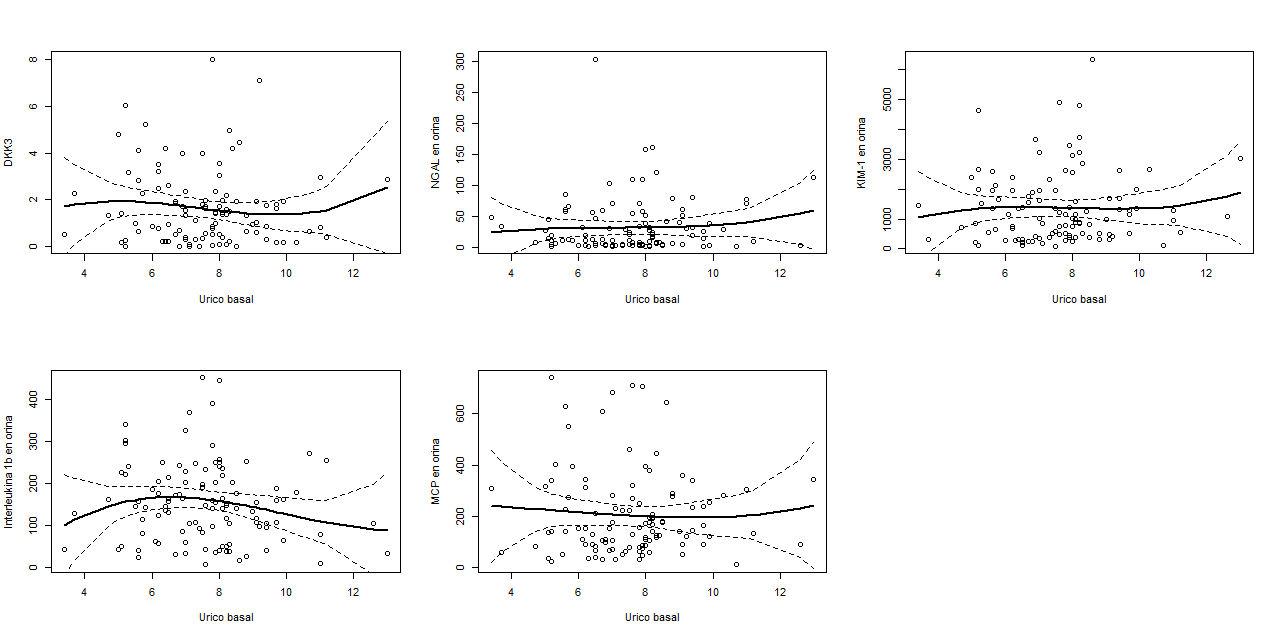


**TABLE. Relationship between BASAL URIC ACID and different markers of renal and tubular damage. Generalized additive regression (GAM) models adjusting for age, proteinuria (log-transformed), residual renal function, diabetes and statin or angiotensin-converting enzyme inhibitors treatment.**

|  | **DKK3 (log)** | | | | **NGAL(log)** | | | | **KIM-1(log)** | | | | **IL1B_B(log)** | | | | **MCP(log)** | | |
| --- | --- | --- | --- | --- | --- | --- | --- | --- | --- | --- | --- | --- | --- | --- | --- | --- | --- | --- | --- |
|  | **B** | **SE** | **p** | **B** | | **SE** | **p** | **B** | | **SE** | **p** | **B** | | **SE** | **p** | **B** | | **SE** | **p** |
| (Intercept) | 0,291 | 0,556 | 0,603 | 1,642 | | 0,416 | 0,000 | 3,084 | | 0,340 | 0,000 | 0,837 | | 0,397 | 0,045 | 2,512 | | 0,296 | 0,000 |
| **BASAL URIC ACID** | -0,004 | 0,037 | 0,904 | 0,004 | | 0,028 | 0,900 | 0,000 | | 0,023 | 0,984 | -0,008 | | 0,033 | 0,806 | -0,008 | | 0,020 | 0,693 |
| **AGE** | 0,004 | 0,005 | 0,445 | -0,001 | | 0,004 | 0,802 | 0,002 | | 0,003 | 0,490 | 0,003 | | 0,005 | 0,494 | 0,000 | | 0,003 | 0,883 |
| **RESIDUAL RENAL FUNCTION** | -0,011 | 0,009 | 0,239 | -0,009 | | 0,007 | 0,191 | -0,004 | | 0,006 | 0,490 | -0,005 | | 0,007 | 0,462 | -0,003 | | 0,005 | 0,514 |
| **BASAL PROTEINURIA (log-transformed)** |  |  | **0,006** | 0,190 | | 0,038 | **<0,001** | 0,098 | | 0,031 | **0,002** | 0,048 | | 0,044 | 0,287 | 0,101 | | 0,027 | **<0,001** |
| **DIABETES** | -0,213 | 0,125 | 0,091 | 0,017 | | 0,099 | 0,862 | 0,135 | | 0,080 | 0,096 | 0,021 | | 0,093 | 0,822 | 0,099 | | 0,070 | 0,162 |
| **STATIN** | -0,357 | 0,139 | **0,012** | -0,083 | | 0,110 | 0,451 | -0,194 | | 0,090 | **0,033** | 0,007 | | 0,110 | 0,953 | -0,164 | | 0,078 | **0,039** |
| **ACE INHIBITORS** | -0,122 | 0,118 | 0,306 | -0,018 | | 0,094 | 0,848 | -0,068 | | 0,076 | 0,374 | -0,189 | | 0,090 | 0,046 | -0,037 | | 0,067 | 0,582 |

*Introduced as a non-linear tem in the model

**Table 5. Adjusted relationship between plasma uric acid levels and the selected urinary markers of kidney injury**

|  | **DKK3(log)** | | | **NGAL(log)** | | | **KIM-1(log)** | | | **IL1B_B(log)** | | | **MCP(log)** | | |
| --- | --- | --- | --- | --- | --- | --- | --- | --- | --- | --- | --- | --- | --- | --- | --- |
|  | **B** | **SE** | **p** | **B** | **SE** | **p** | **B** | **SE** | **p** | **B** | **SE** | **p** | **B** | **SE** | **p** |
| **(Intercept)** | 0.329 | 0.469 | 0.485 | 1.611 | 0.357 | 0.000 | 3.064 | 0.291 | <2e-16 | 0.632 | 0.369 | 0.101 | 2.482 | 0.255 | 0.000 |
| **Plasma uric acid (ref. 1^st^ quartile)** |  |  |  |  |  |  |  |  |  |  |  |  |  |  |  |
| 2^nd^ quartile | -0.224 | 0.160 | 0.165 | -0.009 | 0.126 | 0.944 | -0.053 | 0.102 | 0.608 | 0.200 | 0.126 | 0.128 | -0.031 | 0.090 | 0.728 |
| 3^rd^ quartile | -0.052 | 0.172 | 0.762 | 0.096 | 0.134 | 0.474 | 0.077 | 0.109 | 0.482 | 0.039 | 0.134 | 0.776 | -0.071 | 0.095 | 0.457 |
| 4^th^ quartile | -0.025 | 0.168 | 0.884 | 0.069 | 0.132 | 0.599 | -0.029 | 0.107 | 0.789 | 0.077 | 0.165 | 0.644 | -0.009 | 0.094 | 0.927 |
| **Age (years)** | 0.004 | 0.005 | 0.424 | 0.000 | 0.004 | 0.932 | 0.003 | 0.004 | 0.373 | 0.004 | 0.005 | 0.379 | -0.001 | 0.003 | 0.825 |
| **GFR (mL/min)** | -0.011 | 0.009 | 0.226 | -0.011 | 0.007 | 0.146 | -0.006 | 0.006 | 0.355 | -0.004 | 0.007 | 0.608 | -0.003 | 0.005 | 0.631 |
| **Proteinuria (log)** |  |  | **0.007** | 0.188 | 0.038 | **<0.001** | 0.096 | 0.031 | **0.003** | 0.041 | 0.043 | 0.351 | 0.101 | 0.027 | **<0.001** |
| **Diabetes** | -0.227 | 0.126 | 0.075 | 0.012 | 0.100 | 0.906 | 0.144 | 0.081 | 0.078 | 0.027 | 0.106 | 0.805 | 0.087 | 0.071 | 0.221 |
| **Statin therapy** | -0.357 | 0.139 | **0.012** | -0.090 | 0.111 | 0.422 | -0.198 | 0.090 | **0.031** | -0.023 | 0.113 | 0.840 | -0.161 | 0.079 | **0.045** |
| **RAA antagonists** | -0.106 | 0.117 | 0.366 | -0.011 | 0.093 | 0.909 | -0.063 | 0.076 | 0.411 | -0.206 | 0.089 | 0.031 | -0.034 | 0.066 | 0.608 |

Generalized additive regression models (GAM), adjusting for age, proteinuria (log-transformed), residual kidney function, diabetes and statin or angiotensin converting enzyme inhibitors treatment.

GFR: Glomerular filtration rate; RAA: Renin-angiotensin axis

# **ANÁLISIS DEL ÚRICO EN ORINA DE 24 H BASAL**

**TABLE. Relationship between URINARY URIC ACID IN 24 HOURS and different markers of renal and tubular damage. Generalized additive regression (GAM) models adjusting for age, proteinuria (log-transformed), residual renal function, diabetes and statin or angiotensin-converting enzyme inhibitors treatment.**

|  | **DKK3 (log)** | | | **NGAL(log)** | | | **KIM-1(log)** | | | **IL1B_B(log)** | | | **MCP(log)** | | |
| --- | --- | --- | --- | --- | --- | --- | --- | --- | --- | --- | --- | --- | --- | --- | --- |
|  | **B** | **SE** | **p** | **B** | **SE** | **p** | **B** | **SE** | **p** | **B** | **SE** | **p** | **B** | **SE** | **p** |
| (Intercept) | 0,022 | 0,440 | 0,960 | 1,522 | 0,166 | 0,000 | 3,090 | 0,285 | <0,001 | 0,928 | 0,337 | 0,011 | 2,474 | 0,250 | <0,001 |
| **URINARY URIC ACID IN 24 HOURS** | 0,002 | 0,001 | **0,006** | 0,001 | 0,000 | **0,016** | 0,000 | 0,000 | 0,888 | 0,001 | 0,000 | 0,076 | 0,000 | 0,000 | 0,137 |
| **AGE** | 0,007 | 0,005 | 0,189 |  |  | 0,275 | 0,002 | 0,003 | 0,505 | 0,002 | 0,004 | 0,611 | -0,001 | 0,003 | 0,705 |
| **RESIDUAL RENAL FUNCTION** | -0,031 | 0,011 | **0,006** | -0,021 | 0,009 | **0,021** | -0,003 | 0,008 | 0,657 | -0,019 | 0,009 | 0,049 | 0,002 | 0,006 | 0,797 |
| **BASAL PROTEINURIA (log-transformed)** |  |  | **0,021** | 0,176 | 0,037 | **<0,001** | 0,099 | 0,032 | **0,002** | 0,013 | 0,039 | 0,742 |  |  | **<0,001** |
| **DIABETES** | -0,236 | 0,118 | **0,049** | -0,001 | 0,094 | 0,989 | 0,137 | 0,079 | 0,088 | -0,014 | 0,084 | 0,871 | 0,101 | 0,068 | 0,140 |
| **STATIN TREATMENT** | -0,339 | 0,133 | **0,013** | -0,072 | 0,106 | 0,499 | -0,194 | 0,090 | **0,033** | -0,028 | 0,096 | 0,774 | -0,169 | 0,077 | **0,030** |
| **RAA antagonists** | -0,103 | 0,112 | 0,359 | -0,023 | 0,088 | 0,798 | -0,069 | 0,075 | 0,361 | -0,170 | 0,084 | 0,055 | -0,043 | 0,064 | 0,507 |

*Introduced as a non-linear tem in the model

**Table 6. Adjusted relationship between total urinary uric acid excretion and the selected urinary markers of kidney injury**

|  | **DKK3(log)** | | | **NGAL(log)** | | | **KIM-1(log)** | | | | **IL1B_B(log)** | | | | **MCP(log)** | | |
| --- | --- | --- | --- | --- | --- | --- | --- | --- | --- | --- | --- | --- | --- | --- | --- | --- | --- |
|  | **B** | **SE** | **p** | **B** | **SE** | **p** | **B** | **SE** | **p** | **B** | | **SE** | **p** | **B** | | **SE** | **p** |
| **(Intercept)** | 0.230 | 0.445 | 0.606 | 1.596 | 0.177 | 0.000 | 3.085 | 0.283 | <0.001 | 0.980 | | 0.375 | 0.016 | 2.414 | | 0.249 | 0.000 |
| **Total urinary uric acid excretion (ref. 1^st^ quartile)** |  |  |  |  |  |  |  |  |  |  | |  |  |  | |  |  |
| 2^nd^ quartile | 0.156 | 0.163 | 0.341 | -0.005 | 0.129 | 0.969 | 0.027 | 0.107 | 0.800 | 0.257 | | 0.153 | 0.108 | 0.089 | | 0.091 | 0.335 |
| 3^rd^ quartile | -0.003 | 0.162 | 0.985 | 0.124 | 0.128 | 0.332 | 0.079 | 0.106 | 0.459 | 0.049 | | 0.137 | 0.722 | 0.035 | | 0.091 | 0.699 |
| 4^th^ quartile | 0.399 | 0.204 | 0.053 | 0.208 | 0.157 | 0.189 | -0.084 | 0.131 | 0.523 | 0.185 | | 0.172 | 0.296 | -0.112 | | 0.112 | 0.318 |
| **Age (years)** | 0.006 | 0.005 | 0.245 |  |  | 0.263 | 0.002 | 0.003 | 0.655 | 0.001 | | 0.005 | 0.787 | -0.002 | | 0.003 | 0.608 |
| **GFR (mL/min)** | -0.025 | 0.011 | **0.027** | -0.014 | 0.009 | 0.111 | -0.001 | 0.007 | 0.903 | -0.013 | | 0.010 | 0.202 | 0.000 | | 0.006 | 0.985 |
| **Proteinuria (log)** |  |  | **0.009** | 0.184 | 0.038 | **<0.001** | 0.096 | 0.032 | **0.003** | 0.018 | | 0.042 | 0.673 |  | |  | **0.001** |
| **Diabetes** | -0.217 | 0.122 | 0.079 | 0.000 | 0.098 | 0.998 | 0.139 | 0.080 | 0.086 | 0.026 | | 0.087 | 0.767 | 0.105 | | 0.068 | 0.128 |
| **Statin therapy** | -0.338 | 0.138 | **0.016** | -0.067 | 0.109 | 0.540 | -0.200 | 0.090 | **0.029** | -0.039 | | 0.108 | 0.721 | -0.175 | | 0.077 | **0.025** |
| **RAA antagonists** | -0.097 | 0.115 | 0.401 | -0.023 | 0.091 | 0.799 | -0.079 | 0.076 | 0.300 | -0.182 | | 0.092 | 0.059 | -0.048 | | 0.065 | 0.459 |

Generalized additive regression models (GAM), adjusting for age, proteinuria (log-transformed), residual kidney function, diabetes and statin or angiotensin converting enzyme inhibitors treatment.

GFR: Glomerular filtration rate; RAA: Renin-angiotensin axis

# **ANÁLISIS DE LA CONCENTRACIÓN DE ÚRICO EN ORINA BASAL**

**TABLE. Relationship between URIC ACID CONCENTRATION IN URINE and different markers of renal and tubular damage. Generalized additive regression (GAM) models adjusting for age, proteinuria (log-transformed), residual renal function, diabetes and statin or angiotensin-converting enzyme inhibitors treatment.**

|  | **DKK3 (log)** | | | | **NGAL(log)** | | | | **KIM-1(log)** | | | **IL1B_B(log)** | | | | **MCP(log)** | | | |
| --- | --- | --- | --- | --- | --- | --- | --- | --- | --- | --- | --- | --- | --- | --- | --- | --- | --- | --- | --- |
|  | **B** | **SE** | **p** | **B** | | **SE** | **p** | **B** | | **SE** | **p** | **B** | **SE** | **p** | **B** | | **SE** | **p** |  |
| (Intercept) | 0,468 | 0,446 | 0,297 | 1,681 | | 0,176 | <0,001 | 3,083 | | 0,280 | <0,001 | 1,044 | 0,316 | 0,004 | 2,418 | | 0,254 | <0,001 |  |
| **URIC ACID CONCENTRATION IN URINE** |  |  | **0,020** |  | |  | **0,040*** | 0,007 | | 0,007 | 0,293 |  |  | **0,017*** |  | |  | 0,321* |  |
| **AGE** | 0,004 | 0,005 | 0,467 |  | |  | 0,170 | 0,002 | | 0,003 | 0,583 | 0,003 | 0,004 | 0,459 | 0,000 | | 0,003 | 0,909 |  |
| **RESIDUAL RENAL FUNCTION** | -0,022 | 0,009 | **0,023** | -0,015 | | 0,008 | **0,049** | -0,007 | | 0,007 | 0,270 | -0,017 | 0,007 | **0,025** | -0,005 | | 0,006 | 0,391 |  |
| **BASAL PROTEINURIA (log-transformed)** |  |  | **0,004*** | 0,188 | | 0,036 | **<0,001*** | 0,095 | | 0,031 | **0,003** | 0,051 | 0,030 | 0,100 | 0,101 | | 0,026 | **<0,001** |  |
| **DIABETES** | -0,288 | 0,118 | **0,017** | -0,032 | | 0,094 | 0,731 | 0,120 | | 0,080 | 0,135 | -0,091 | 0,073 | 0,224 | 0,085 | | 0,069 | 0,221 |  |
| **STATIN TREATMENT** | -0,293 | 0,132 | **0,029** | -0,031 | | 0,105 | 0,770 | -0,185 | | 0,090 | **0,042** | 0,040 | 0,084 | 0,639 | -0,154 | | 0,077 | **0,049** |  |
| **ACE INHIBITORS TREATMENT** | -0,100 | 0,110 | 0,364 | -0,020 | | 0,086 | 0,816 | -0,066 | | 0,075 | 0,378 | -0,147 | 0,071 | 0,051 | -0,026 | | 0,064 | 0,688 |  |

*Introduced as a non-linear tem in the model

**Table 7. Adjusted relationship between urinary uric acid concentration and the selected urinary markers of kidney injury**

|  | **DKK3(log)** | | | **NGAL(log)** | | | **KIM-1(log)** | | | **IL1B_B(log)** | | | **MCP(log)** | | |
| --- | --- | --- | --- | --- | --- | --- | --- | --- | --- | --- | --- | --- | --- | --- | --- |
|  | **B** | **SE** | **p** | **B** | **SE** | **p** | **B** | **SE** | **p** | **B** | **SE** | **p** | **B** | **SE** | **p** |
| **(Intercept)** | 0.236 | 0.459 | 0.608 | 1.610 | 0.172 | <0.001 | 3.072 | 0.286 | <0.001 | 0.950 | 0.336 | 0.010 | 2.404 | 0.254 | 0.000 |
| **Urinary uric acid concentration (ref. 1^st^ quartile)** |  |  |  |  |  |  |  |  |  |  |  |  |  |  |  |
| 2^nd^ quartile | 0.247 | 0.158 | 0.121 | 0.097 | 0.121 | 0.423 | 0.196 | 0.102 | 0.057 | 0.078 | 0.117 | 0.510 | 0.140 | 0.090 | 0.124 |
| 3^rd^ quartile | 0.263 | 0.155 | 0.094 | 0.039 | 0.120 | 0.749 | 0.041 | 0.100 | 0.684 | -0.114 | 0.122 | 0.358 | 0.060 | 0.089 | 0.503 |
| 4^th^ quartile | 0.397 | 0.185 | **0.035** | 0.369 | 0.142 | **0.011** | 0.217 | 0.119 | 0.071 | 0.278 | 0.148 | 0.073 | 0.107 | 0.105 | 0.310 |
| **Age (years)** | 0.004 | 0.**005** | 0.496 |  |  | 0.266 | 0.002 | 0.003 | 0.481 | 0.003 | 0.004 | 0.503 | 0.000 | 0.003 | 0.975 |
| **GFR (mL/min)** | -0.019 | 0.010 | 0.056 | -0.016 | 0.008 | **0.046** | -0.008 | 0.006 | 0.196 | -0.017 | 0.008 | **0.043** | -0.006 | 0.006 | 0.320 |
| **Proteinuria (log)** |  |  | **0.011** | 0.184 | 0.037 | **<0.001** | 0.091 | 0.031 | **0.004** | 0.037 | 0.035 | 0.300 | 0.097 | 0.027 | **0.001** |
| **Diabetes** | -0.256 | 0.126 | **0.045** | -0.052 | 0.098 | 0.594 | 0.085 | 0.081 | 0.299 | -0.070 | 0.085 | 0.417 | 0.072 | 0.072 | 0.318 |
| **Statin therapy** | -0.349 | 0.137 | **0.013** | -0.067 | 0.106 | 0.526 | -0.192 | 0.088 | **0.032** | 0.061 | 0.097 | 0.538 | -0.166 | 0.078 | **0.037** |
| **RAA antagonists** | -0.106 | 0.115 | 0.357 | -0.025 | 0.088 | 0.777 | -0.067 | 0.074 | 0.367 | -0.178 | 0.081 | **0.038** | -0.032 | 0.065 | 0.624 |

Generalized additive regression models (GAM), adjusting for age, proteinuria (log-transformed), residual kidney function, diabetes and statin or angiotensin converting enzyme inhibitors treatment.

GFR: Glomerular filtration rate; RAA: Renin-angiotensin axis

# **ANÁLISIS DEL ACLARAMIENTO DE ÁCIDO ÚRICO BASAL**

**TABLE. Relationship between URIC ACID CLEARANCE and different markers of renal and tubular damage. Generalized additive regression (GAM) models adjusting for age, proteinuria (log-transformed), residual renal function, diabetes and statin or angiotensin-converting enzyme inhibitors treatment.**

|  | **DKK3 (log)** | | | | **NGAL(log)** | | | | **KIM-1(log)** | | | | **IL1B_B(log)** | | | | **MCP(log)** | | | |
| --- | --- | --- | --- | --- | --- | --- | --- | --- | --- | --- | --- | --- | --- | --- | --- | --- | --- | --- | --- | --- |
|  | **B** | **SE** | **p** | **B** | | **SE** | **p** | **B** | | **SE** | **p** | **B** | | **SE** | **p** | **B** | | **SE** | **p** |  |
| (Intercept) | 0,087 | 0,438 | 0,843 | 1,492 | | 0,167 | <0,001 | 3,090 | | 0,284 | <0,001 | 0,927 | | 0,323 | 0,008 | 2,479 | | 0,246 | 0,000 |  |
| **Urinary uric acid clearanceANCE** | 0,167 | 0,061 | **0,007** | 0,116 | | 0,048 | **0,017** | -0,002 | | 0,041 | 0,956 | 0,095 | | 0,041 | **0,029** | -0,036 | | 0,035 | 0,317 |  |
| **AGE** | 0,005 | 0,005 | 0,289 |  | |  | 0,264* | 0,002 | | 0,003 | 0,491 | 0,002 | | 0,004 | 0,705 | 0,000 | | 0,003 | 0,868 |  |
| **RESIDUAL RENAL FUNCTION** | -0,030 | 0,011 | **0,009** | -0,020 | | 0,009 | **0,023** | -0,004 | | 0,007 | 0,612 | -0,021 | | 0,008 | **0,022** | 0,000 | | 0,006 | 0,946 |  |
| **BASAL PROTEINURIA (log-transformed)** |  |  | **0,020*** | 0,176 | | 0,037 | **<0,001** | 0,099 | | 0,032 | **0,002** | 0,023 | | 0,035 | 0,518 | 0,106 | | 0,027 | **<0,001** |  |
| **DIABETES** | -0,203 | 0,118 | 0,090 | 0,028 | | 0,094 | 0,764 | 0,135 | | 0,079 | 0,089 | 0,007 | | 0,080 | 0,926 | 0,094 | | 0,068 | 0,174 |  |
| **STATIN TREATMENT** | -0,334 | 0,134 | **0,014** | -0,064 | | 0,106 | 0,548 | -0,194 | | 0,090 | **0,033** | 0,002 | | 0,092 | 0,984 | -0,170 | | 0,078 | **0,032** |  |
| **ACE INHIBITORS TREATMENT** | -0,130 | 0,112 | 0,249 | -0,042 | | 0,088 | 0,631 | -0,068 | | 0,075 | 0,366 | -0,169 | | 0,081 | **0,048** | -0,029 | | 0,065 | 0,662 |  |

*Introduced as a non-linear tem in the model

**Table 8. Adjusted relationship between urinary uric acid clearance and the selected urinary markers of kidney injury**

|  | **DKK3(log)** | | | **NGAL(log)** | | | **KIM-1(log)** | | | **IL1B_B(log)** | | | **MCP(log)** | | |
| --- | --- | --- | --- | --- | --- | --- | --- | --- | --- | --- | --- | --- | --- | --- | --- |
|  | **B** | **SE** | **p** | **B** | **SE** | **p** | **B** | **SE** | **p** | **B** | **SE** | **p** | **B** | **SE** | **p** |
| **(Intercept)** | 0.349 | 0.442 | 0.432 | 1.566 | 0.177 | <0.001 | 3.114 | 0.287 | 0.000 | 1.096 | 0.352 | 0.005 | 2.438 | 0.250 | 0.000 |
| **Urinary uric acid clearance (ref. 1^st^ quartile)** |  |  |  |  |  |  |  |  |  |  |  |  |  |  |  |
| 2^nd^ quartile | 0.148 | 0.155 | 0.341 | 0.125 | 0.125 | 0.317 | 0.053 | 0.105 | 0.612 | 0.158 | 0.120 | 0.201 | 0.023 | 0.092 | 0.799 |
| 3^rd^ quartile | 0.130 | 0.188 | 0.492 | 0.216 | 0.141 | 0.129 | -0.027 | 0.120 | 0.822 | -0.178 | 0.190 | 0.361 | -0.007 | 0.104 | 0.949 |
| 4^th^ quartile | 0.513 | 0.189 | **0.008** | 0.295 | 0.147 | **0.048** | 0.048 | 0.124 | 0.701 | 0.187 | 0.152 | 0.233 | -0.035 | 0.108 | 0.747 |
| **Age (years)** | 0.005 | 0.005 | 0.373 |  |  | 0.249* | 0.002 | 0.003 | 0.550 | -0.001 | 0.005 | 0.834 | 0.000 | 0.003 | 0.881 |
| **GFR (mL/min)** | -0.028 | 0.011 | **0.010** | -0.017 | 0.008 | 0.051 | -0.005 | 0.007 | 0.453 | -0.010 | 0.009 | 0.248 | -0.002 | 0.006 | 0.720 |
| **Proteinuria (log)** |  |  | **0.029*** | 0.172 | 0.039 | **<0.001** | 0.100 | 0.033 | **0.003** | 0.035 | 0.041 | 0.393 | 0.103 | 0.029 | **0.001** |
| **Diabetes** | -0.167 | 0.121 | 0.169 | 0.037 | 0.097 | 0.699 | 0.143 | 0.080 | 0.076 | -0.004 | 0.081 | 0.959 | 0.093 | 0.070 | 0.186 |
| **Statin therapy** | -0.369 | 0.135 | **0.007** | -0.080 | 0.108 | 0.459 | -0.198 | 0.091 | **0.031** | 0.009 | 0.099 | 0.926 | -0.164 | 0.079 | **0.041** |
| **RAA antagonists** | -0.106 | 0.113 | 0.350 | -0.034 | 0.089 | 0.704 | -0.066 | 0.076 | 0.384 | -0.240 | 0.090 | 0.014 | -0.033 | 0.066 | 0.616 |

Generalized additive regression models (GAM), adjusting for age, proteinuria (log-transformed), residual kidney function, diabetes and statin or angiotensin converting enzyme inhibitors treatment.

*Introduced as a non-linear tem in the model

GFR: Glomerular filtration rate; RAA: Renin-angiotensin axis

# **ANÁLISIS DE LA EXCRECIÓN FRACCIONADA DE ÚRICO**

**Figura. Smoothing regression showing the relationship between fractional excretion of uric acid and**  **markers of renal and tubular damage**.


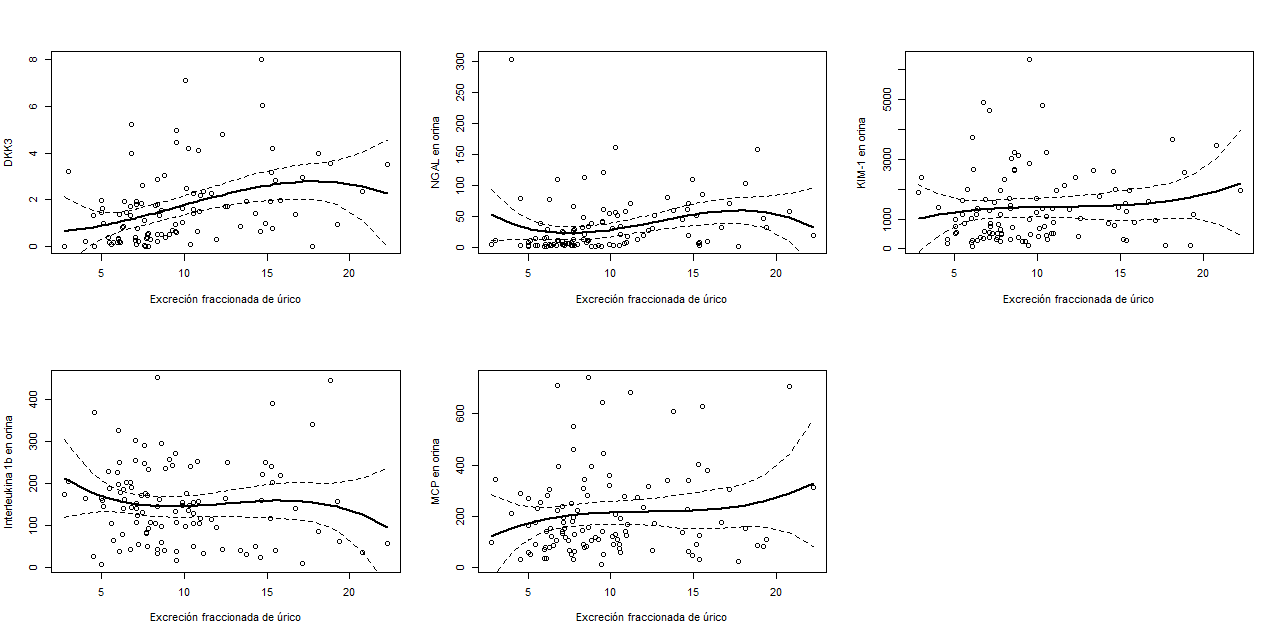


**Table. Relationship between fractional excretion of uric acid and different markers of renal and tubular damage. Generalized additive regression (GAM) models adjusting for age, proteinuria (log-transformed), función renal residual, diabetes y tratamiento con estatinas o IECAS.**

|  | **DKK3** | | | **NGAL** | | | **KIM-1** | | | | **IL1B_B** | | | | **MCP** | | | |  |
| --- | --- | --- | --- | --- | --- | --- | --- | --- | --- | --- | --- | --- | --- | --- | --- | --- | --- | --- | --- |
|  | **B** | **SE** | **p** | **B** | **SE** | **p** | | **B** | **SE** | **p** | | **B** | **SE** | **p** | | **B** | **SE** | **p** | |
| (Intercept) | 0,148 | 0,431 | 0,732 | 1,524 | 0,165 | 0,000 | | 3,096 | 0,303 | 0,000 | | 0,735 | 0,323 | 0,032 | | 2,512 | 0,264 | 0,000 | |
| **FRACTIONAL EXCRETION OF URIC ACID** |  |  | **0,014*** |  |  | 0,089 | | -0,001 | 0,010 | 0,946 | | 0,024 | 0,011 | **0,040** | | -0,006 | 0,008 | 0,500 | |
| **AGE** | 0,005 | 0,005 | 0,317 |  |  | 0,232 | | 0,002 | 0,003 | 0,488 | | 0,001 | 0,004 | 0,783 | | 0,000 | 0,003 | 0,917 | |
| **RESIDUAL RENAL FUNCTION** | -0,010 | 0,009 | 0,256 | -0,007 | 0,007 | 0,308 | | -0,004 | 0,006 | 0,486 | | -0,008 | 0,006 | 0,158 | | -0,004 | 0,005 | 0,441 | |
| **BASAL PROTEINURIA (log-transformed)** |  |  | **0,030*** | 0,177 | 0,038 | **0,000** | | 0,099 | 0,032 | 0,003 | | 0,015 | 0,037 | 0,689 | | 0,106 | 0,028 | **0,000** | |
| **DIABETES** | -0,185 | 0,118 | 0,120 | 0,061 | 0,095 | 0,522 | | 0,135 | 0,079 | 0,089 | | 0,023 | 0,081 | 0,774 | | 0,091 | 0,069 | 0,185 | |
| **STATIN TREATMENT** | -0,338 | 0,133 | **0,012** | -0,076 | 0,106 | 0,477 | | -0,194 | 0,090 | **0,033** | | -0,004 | 0,093 | 0,964 | | -0,166 | 0,078 | **0,036** | |
| **ACE INHIBITORS TREATMENT** | -0,120 | 0,111 | 0,284 | -0,029 | 0,087 | 0,741 | | -0,068 | 0,075 | 0,366 | | -0,173 | 0,082 | **0,046** | | -0,030 | 0,065 | 0,652 | |

*Introduced as a non-linear tem in the model

**Table 9. Adjusted relationship between fractional urinary excretion of uric acid and the selected urinary markers of kidney injury**

|  | **DKK3** | | | **NGAL** | | | **KIM-1** | | | **IL1B_B** | | | | **MCP** | | |
| --- | --- | --- | --- | --- | --- | --- | --- | --- | --- | --- | --- | --- | --- | --- | --- | --- |
|  | **B** | **SE** | **p** | **B** | **SE** | **p** | **B** | **SE** | **p** | | **B** | **SE** | **p** | **B** | **SE** | **p** |
| **(Intercept)** | -0.033 | 0.455 | 0.942 | 1.375 | 0.194 | 0.000 | 3.056 | 0.307 | 0.000 | | 1.120 | 0.315 | 0.002 | 2.414 | 0.267 | 0.000 |
| **Fractional excretion of uric acid (ref. 1^st^ quartile)** |  |  |  |  |  |  |  |  |  | |  |  |  |  |  |  |
| 2^nd^ quartile | 0.046 | 0.155 | 0.769 | 0.075 | 0.125 | 0.552 | 0.023 | 0.107 | 0.833 | | -0.215 | 0.112 | 0.068 | 0.060 | 0.093 | 0.520 |
| 3^rd^ quartile | 0.443 | 0.160 | **0.007** | 0.220 | 0.129 | 0.093 | 0.008 | 0.111 | 0.941 | | 0.006 | 0.101 | 0.955 | 0.028 | 0.096 | 0.770 |
| 4^th^ quartile | 0.398 | 0.164 | **0.017** | 0.309 | 0.133 | **0.022** | 0.031 | 0.114 | 0.789 | | 0.225 | 0.111 | 0.055 | -0.004 | 0.099 | 0.967 |
| **Age (years)** | 0.005 | 0.005 | 0.307 |  |  | 0.282* | 0.003 | 0.003 | 0.471 | | 0.000 | 0.004 | 0.981 | 0.000 | 0.003 | 0.966 |
| **GFR (mL/min)** | -0.013 | 0.009 | 0.148 | -0.007 | 0.007 | 0.316 | -0.004 | 0.006 | 0.527 | | -0.010 | 0.006 | 0.079 | -0.004 | 0.005 | 0.459 |
| **Proteinuria(log)** |  |  | **0.023*** | 0.172 | 0.038 | **0.000** | 0.096 | 0.032 | **0.004** | | 0.001 | 0.033 | 0.975 | 0.103 | 0.028 | 0.000 |
| **Diabetes** | -0.172 | 0.120 | 0.155 | 0.043 | 0.096 | 0.658 | 0.135 | 0.081 | 0.101 | | 0.065 | 0.074 | 0.389 | 0.081 | 0.071 | 0.256 |
| **Statin therapy** | -0.322 | 0.133 | **0.018** | -0.069 | 0.108 | 0.523 | -0.194 | 0.092 | **0.038** | | -0.050 | 0.085 | 0.567 | -0.157 | 0.080 | 0.053 |
| **RAA antagonists** | -0.121 | 0.112 | 0.280 | -0.030 | 0.089 | 0.735 | -0.067 | 0.077 | 0.387 | | -0.213 | 0.076 | **0.011** | -0.026 | 0.067 | 0.693 |

Generalized additive regression models (GAM), adjusting for age, proteinuria (log-transformed), residual kidney function, diabetes and statin or angiotensin converting enzyme inhibitors treatment.

*Introduced as a non-linear tem in the model

GFR: Glomerular filtration rate; RAA: Renin-angiotensin axis
