# Supplementary material for: Association of urinary excretion rates of uric acid with biomarkers of kidney injury in patients with advanced chronic kidney disease
Source: PLoS One. 2024 Jun 11;19(6):e0304105. doi: 10.1371/journal.pone.0304105 (PMC11166352; doi:10.1371/journal.pone.0304105)
Supplement: Attachment — Submitted filename: Rebuttal_Letter_PLOSONE.docx [file pone.0304105.s007.docx]

Dear Editor,

Thank you for your positive evaluation of our manuscript “Association of urinary excretion rates of uric acid with biomarkers of kidney injury in patients with advanced chronic kidney disease” (PONE D-24-4504). We are submitting a revised version of the paper, in which we have done our best to follow the recommendations of the reviewers and the Editorial Board. We hope that this new version may be satisfactory, but will consider with pleasure any additional request.

We include a marked-up manuscript, with the modifications highlighted in red, as also an unmarked version of the paper. We have reviewed the manuscript, looking for typographic errors. We have no further allegations concerning the Journal requirements. Please, let us know if there is still something wrong or equivocal.

We shall answer now in detail to the requests o the reviewers:

Reviewer #1

We thank the reviewer for his/her kind comments. We have made the six text corrections suggested

Reviewer #2

Again, thank you for your kind comments.

- We have included a paragraph in the Method chapter, to define asymptomatic hiperuricemia. Patients with active or recent (<12 months) gouty arthritis or uric litiasis were excluded (n=2 patients, included in Figure 1 as “comorbidity” cases)
- We have underlined in Results the fact that only 4th quartile levels of UA excretion showed a consistent association with markers of injury
- Yes, the reviewer is right!. Figures 3 and 4 are the same. We include now real Figure 4. We apologize for the mistake
- We include now urinary creatinine level (Table 3)
- We have extended our comments in the Results although, in general, we preferred to leave this for the Discussion
- We have corrected the indicated typographic errors
- We have improved the consistency of the figures, making pertinent changes (Tables 5 to 9)
- We include now the codes of the ELISA kits
- We have added the formulas for estimation of urea, creatinine and UA clearances.

Again, thank you for your help. We await Word from your ditorial Board. Any new request will be considered.

Sincerely

Miguel Pérez Fontán

Corresponding author
